# Development of continuing professional development standards for Allied Healthcare Professionals in Pakistan

**DOI:** 10.12669/pjms.41.10.11585

**Published:** 2025-10

**Authors:** Shazia Tazion, Usman Mahboob, Kinza Aslam, Rehan Ahmed Khan

**Affiliations:** 1 Shazia Tazion Associate Professor (Obstetrics & Gynaecology), Sharif Medical and Dental College Lahore Pakistan; 2 Usman Mahboob Institute of Health Professions Education and Research, Khyber Medical University Peshawar, Pakistan; 3 Kinza Aslam Associate Director (Medical Education) University of Lahore, Pakistan; 4 Rehan Ahmed Khan Riphah Institute of Assessment, Riphah International University, Rawalpindi, Pakistan

**Keywords:** CPD Standards, Evidence-based Practices, Healthcare Professionals, Pakistan, Patient Safety, Professional Competence

## Abstract

**Objectives:**

To explore health professionals’ perceptions regarding the development of Continuing Professional Development (CPD) standards for allied health professionals (AHP) in Pakistan and to develop these CPD standards.

**Methodology:**

A three rounds Delphi study was conducted at the University of Lahore from January 2024 to June 2024 to develop CPD standards for allied health professionals. Semi-structured interviews and iterative questionnaires gathered health professionals’ views on core competencies, barriers, and solutions. Expert opinions were validated and aligned with WFME standards to produce the final proposed standards.

**Results:**

Fourteen participants who were followed in rounds two and three were interviewed. Six themes emerged from the first round: CPD standards, competency development, barriers, interprofessional education, allied health professionals, and assessment. Key qualities identified included clinical experience, communication skills, and cultural sensitivity, while time and budget were noted as challenges. In round two, 26 standards were generated, all showing consistency in median and IQR analysis. Round three showed only a minor shift in agreement on one statement. Final CPD standards were developed through expert consensus, aligned with the WFME framework, and validated by the expert panel for credibility

**Conclusion:**

The study highlights the need for collaboration, evidence based practices, and ongoing research to enhance patient care and professional growth. The proposed CPD standards for allied health professionals in Pakistan through expert input are based on consideration of the local context and learning needs.

***Abbreviations:* CPD:** Continuing professional development, **AHP:** Allied healthcare professionals, **IPE:** Interprofessional education, **WFME:** World Federation of Medical Education, **WHO:** World Health Organization, **HCPC:** Health Care Professional Council.

## INTRODUCTION

Continuing professional development (CPD) is mandatory for health professionals worldwide to maintain their credentials and ensure their right to practice.^1^ CPD begins after completing basic and postgraduate medical education and continues throughout a professional’s career.^2^ CPD enhances knowledge, skills, and professional proficiency, improving care quality, team standards, service delivery, annual appraisals, career advancement, work satisfaction, and skill development in leadership and education in high income countries.^3^

The World Federation For Medical Education emphasizes the importance of tailored CPD activities, structured learning, and follow-up activities; promoting growth, communication, and change efforts; facilitating research; saving time and money; and driving continuous improvement.^3^ AHP’s, including dental hygienists, sonographers, dieticians, occupational therapists, and others, play crucial roles in illness and disorder identification, assessment, prevention, and management.^4^ Countries such as the UK, Australia, New Zealand, Canada, Qatar, and Bermuda have established CPD standards for their allied health workforce.^5^ However, in low- and lower-middle-income countries, limited opportunities and the inaccessibility of stakeholders hinder the development and implementation of CPD standards.^6^ CPD is essential for maintaining registration and licensure in allied health professions, with regulatory bodies requiring verification.^6^

The Health and Care Professions Council in UK oversees various allied health professions, whereas Allied Health Care Professional Council (AHCP)in Pakistan regulates service standards and provide CPD system.^7^ However, the council has yet to develop CPD standards for allied health professionals, highlighting the need for improved patient outcomes and professionalism. Despite the importance of allied health specialties in Pakistani healthcare system, the focus of continuing professional education is primarily on physicians.^8^,^9^

This study aimed to explore health professionals’ perceptions regarding CPD standard development for allied healthcare professionals in Pakistan and to develop a framework of CPD standards for allied health professionals in Pakistan. These findings will improve healthcare service quality and drive policy making in the local context of Pakistan.

## METHODOLOGY

This Delphi study was conducted at the University of Lahore, Pakistan, from January 2024 to June 2024 to address the unexamined area of CPD standards for allied health professionals (AHP) in the local context. The three-round Delphi process incorporated participant perceptions from each preceding round.

Of 20 invited experts, 14 were purposively selected for their expertise in allied health sciences and medical education. They included Deans, Principals, Allied Health Council members, and medical educationists from Punjab, Khyber Pakhtunkhwa, Azad Jammu Kashmir, Gilgit Baltistan, and federal institutions.

### Ethical approval:

The institutional EC approved this study Ref. (ERC 138/23/12; 12/12/2023; dated December 12, 2023) and informed consent, confidentiality, and anonymity were maintained.

Round one comprised semi-structured interviews via telephone, WhatsApp, or Zoom in Urdu and English, lasting 25–30 minutes. Data underwent thematic analysis and member checking. A questionnaire for round two was developed from these themes, with items rated on a five points Likert scale. Data were analyzed in SPSS (medians, IQRs), and results informed the third-round questionnaire, which included group medians and allowed revisions. Consensus criteria were: median ≥ 4.5, IQR ≤ 1, and ≥ 80% agreement at ratings Four or Five.

Validity was ensured through authenticity, plausibility, neutrality, literature review, expert selection, member checking, and multiple Delphi rounds. The third round achieved consensus, and the final CPD standards, aligned with WFME guidelines, were validated by the expert panel for clarity, relevance, and feasibility.

## RESULTS

All experts (n=14) participated in Delphi round one and two, whereas only one expert could not join Round three despite repeated reminders. The duration was 30, 15, and seven days for Rounds one, two and three respectively. Average age of all participants was 62.1(±40.23) years, 11 (78.5%) were males, and three (21.4%) were females. Table-I shows the demographic information of all the participants.

**Table-I:** Demographic information of the experts

S. No.	Experts	Gender	Qualification	Academic title	Occupation
1	Member 1	M	PhD (Physical therapy)	Principal College of Rehabilitation Sciences	Physical Therapist
2	Member 2	M	MBBS, MHPE	HOD Assistant Professor Medical Education	Medical Educationist
3	Member 3	M	PhD (Physical therapy)	Dean of Allied Health Sciences	Physical Therapist
4	Member 4	F	MBBS, FCPS	Dean Academics. Develop s BS-level program for optometry	Ophthalmologist
5	Member 5	M	MBBS, Diploma in Public Health	Principal Allied Institute in a Government Medical College Lahore	Principal Research Officer
6	Member 6	M	PhD (Physical therapy)	Dean Academics	Physical Therapist
7	Member 7	M	MBBS, MHPE	Assistant professor of Medical Education	Medical educationist
8	Member 8	M	PhD in Rehabilitation Sciences. Member of the Allied Health Council	Associate Professor at the University	Physical Therapist
9	Member 9	F	MBBS. MHPE	Deputy Director of Medical Education	Medical Educationist
10	Member 10	M	BS & MPhil Physiotherapy, Masters in Health Administration, Diploma in Lab Technology	Member of the Allied Council	Chief Technologist Supreme head health employee organization
11	Member 11	M	MBBS, FCPS	Principal Allied Health Professionals in private medical college	Professor of Community Medicine
12	Member 12	F	MBBS, PhD (Anatomy), FCPS (Surgery)	Associate professor, Director of Academics and Admission.	Associate Professor Anatomy
13	Member 13	M	PhD in Molecular Biology and Clinical Genetics	Assistant Professor Member of the Allied Health Council	Assistant professor
14	Member 14	M	Chemical Pathology	Senior Lab Technologist	Senior Lab Technologist

### Delphi Round One Results:

Thematic data analysis identified 120 open codes, 43 codes, and 23 selective codes from participants. The participants emphasized the importance of establishing standardized CPD standards for consistency and quality. The strengths of CPD include skill enhancement, career advancement opportunities, adaptability, and networking opportunities for professional growth and knowledge sharing. The six themes were identified through thematic analysis, along with selective codes and representative quotes (Table-II).

**Table-II:** Themes and representative Quotes from the Delphi expert Group on Developing CPD standards for Allied healthcare professionals in Pakistan.

Themes	Selective codes	Representative Quotes
CPD Standards	Strengths of CPD Weakness of CPD Personalized focus Time constraints	*‘’The strength of CDP includes that it increases personal capacity building, improves skills, upgrades knowledge, provides promotion opportunities, gets incentives, and improves workplace dealing in a better way. It is also for the professional growth of individuals and raises awareness about recent advances in the field.’’ ‘’CPD facilities stay updated on recent advances and get familiar with them, which I consider a significant strength.’’ ‘’Weaknesses about allied health professionals typically include that they are person-specific. Generally, CPD allows individuals to focus on areas where they feel they lack expertise or where they excel.’’*
Competencies & Standard Development	Improve knowledge Culture competencies Critical competencies CPD & competencies	*‘‘Continuing professional development is vital for staying updated and maintaining different competencies. Support from regulatory bodies, educational institutions, professional associations, and healthcare organizations is crucial for fostering these competencies.’’*
Barriers and challenges of CPD	Implementation of CPD Financial constraints Institutional barriers	*The main factor is cost as attending workshops or courses can be expensive, posing a financial barrier for some individuals. ‘’ ‘’Budget and monetary benefits will be the weakness of these programs.’’ ‘’Adaptability is a problem due to the lack of institutional support. As I am additionally working as Principal, otherwise, I am the principal medical officer. This situation is common throughout Punjab, mostly makeshift people are there.’’*
Interprofessional education	Importance of teamwork Lack of IPE system Interprofessional simulation Linkage & quality of healthcare IPE & collaboration	*‘’Emphasize team-based learning approaches within CPD programs, where professionals work together in interdisciplinary teams to analyze clinical scenarios, develop treatment plans, and address patient care issues.’’* *“CPD practices promote evidence-based practices and we come to know what is happening in the world.’’* *‘’Integrate virtual simulation training into CPD programs to provide hands-on learning experiences in a safe and controlled environment, and virtual simulations can replicate complex clinical scenarios, procedures, and patient interactions, allowing professionals to practice and refine their skills without putting patients at risk.’’* *‘’If a radiologist does an MRI or CT scan for a cancer, he or she doesn’t know what type of cell is in that cancer and can only be tested in the lab for the type of tumor. So, it should be interconnected.’’* *’Effective implementation involves creating joint training programs, fostering interdisciplinary case discussions, and encouraging shared learning experiences to enhance teamwork and communication among healthcare professionals.’’*
Allied healthcare professional	Patient Expectations & Professional Competence Patient-centered approach in healthcare Limited access to resources Quality of CPD program	*‘’Implementing IPE within CPD standards can enhance patient care outcomes, improve interdisciplinary relationships, and address complex healthcare challenges more effectively.’’* *‘’CPD standards can contribute to patient-centered care by emphasizing communication skills, cultural competence, and shared decision-making and training should focus on empathy, active listening, and understanding diverse patient perspectives to enhance healthcare professionals’ ability to provide personalized and inclusive care.’’* *‘’Allied healthcare professionals in remote or rural areas may have limited access to CPD resources, including educational institutions, libraries, and internet connectivity.’’* *‘’CPD activities that lack relevance to the specific needs and interests of allied healthcare professionals may fail to engage or motivate participation.’’*
Assessment of CPD activities	Feedback mechanism Evaluation methods Tools & Strategies	*“Should be a suitable assessment for that training. For example, if training is related to knowledge, the assessment tool will be MCQS which should be enough. But if there is some skill delivery, then that assessment tool should be accordingly designed to assess the skill, like OSPE, DOPS, etc.’’* *“If it is a cognitive activity, then the cognitive tools would be applicable if it is the skill base, the psychomotor tools would be available and the same is for whatever tool it would be, which should be easy to use for both assessors and assesses.”*

### Delphi Round Two Results:

A 26-item questionnaire was generated from the analysis of the results of round one. The CPD data indicate a consensus among experts regarding the importance of CPD standards. The median value of the statements (CPD standards in Table-II) ranged from 5.0 to 1.0, indicating the importance of standardized practices for maintaining consistency and quality across various professions and institutions. The core competencies and skills required for allied healthcare professionals include clinical expertise, communication skills, equipment proficiency, cultural sensitivity, ethical principles, safety protocols, technology integration, and commitment to evidence-based practice. The barriers and challenges associated with CPD provision include recognition, time and financial constraints, limited access, lack of support, and institutional constraints. Collaborative learning environments such as virtual simulation, virtual reality, simulation, and skill labs, foster innovation and teamwork, whereas e-learning modules provide adaptable learning prospects. The assessment domain ranged from 4.50-5.0, emphasizing the importance of tailoring assessment methods of CPD activities, implementing a flexible system, utilizing diverse tools, and maintaining electronic records for CPD activities. The median and interquartile range (IQR) along with the percentage agreement for each statement were calculated (Table-III).

### Delphi Round Three Results:

A Round three questionnaire was used to develop a consensus on the importance of CPD standards and their potential benefits for allied healthcare professionals. Most experts maintained their previous ratings, except for one expert who changed their rating based on group consensus.

### Validation Round:

The proposed CPD standards based on expert consensus and aligned with WFME standards were validated by expert panel again for their clarity, relevance and feasibility through dichotomous statement (Yes/ No) statement. Response rate for this round was 64%. (Table-IV).

## DISCUSSION

This Delphi study done by the authors in Pakistan established CPD standards for allied healthcare workers, highlighting their importance for professional development and patient care outcomes. The strengths of CPD include skill enhancement, career progression, networking opportunities, collaboration, and knowledge sharing, these findings are in line with an Indian and Pakistani study.^10^,^11^ Weaknesses of CPD include person specificity and a lack of accessibility to quality activities. The study done by Shah N et al. from Pakistan and Cunningham DE et al. of Scotland on GP, Pharmacist and nurses also highlighted the need for regular review and adaptability to changing needs aligned with previous research.^12^,^13^ In our study, the experts agreed on the consistency and quality of the CPD standards across different professions and institutions, with 94% agreeing on the need for standardization. It should be used for licensing renewal, with the minimum CPD activity requirement mandated by regulators receiving 89% agreement. This lesser agreement may be due to financial considerations, as CPD acquisition in Pakistan is not government-funded. Therefore, keeping it mandatory for licensing renewal may lead to problems for AHP due to affordability issues. These findings contrast with those from the UK and Australia, where CPD standards are mandatory for license renewal.^14^,^15^

**Fig.1:**
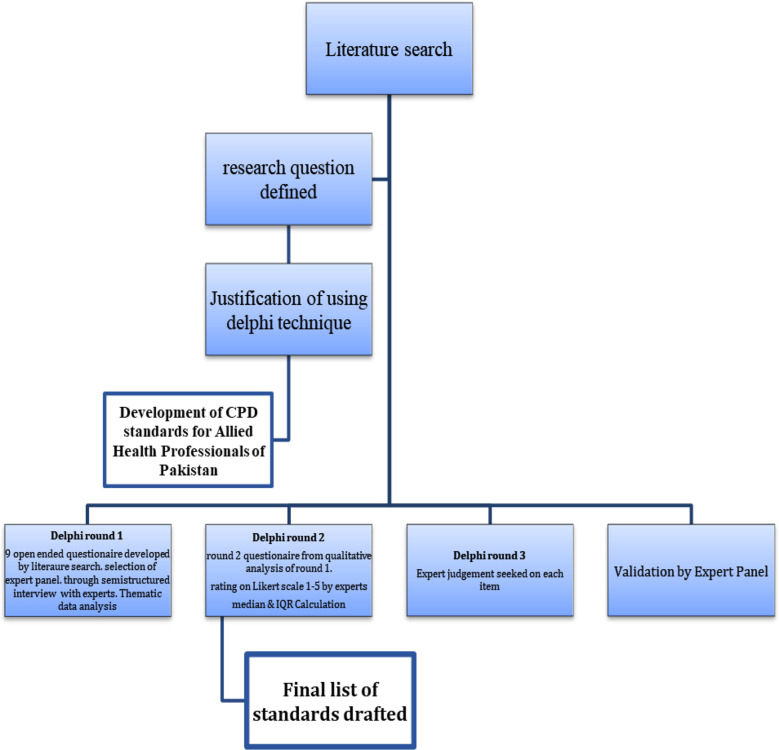
Flow chart of the Delphi rounds.

The study also highlighted the need for virtual platforms, e-learning modules, and simulation technology for skill acquisition and enhancement. These findings were supported by a previous research.^16^ According to the scoping review on allied health professionals in UK by Leone E et al. and a systemic review done in Australia by Main PA et al., CPD is crucial for keeping knowledge up-to-date and remaining aware of recent advances in the field, as suggested by the literature.^17^,^18^

The study identifies competencies such as knowledge, clinical skills, communication skills, critical thinking, decision-making abilities, problem-solving skills, research-oriented competencies, cultural sensitivity, equipment proficiency, safety protocols, and technology integration as essential for professional development. These competencies, acquired through CPD programs, align with previous research emphasizing the importance of CPD standards in promoting professionalism and maintaining competence.^19^ It is crucial to target on-the-job training opportunities and focus on the specific needs of the profession. These findings are in line with a study done by Snowdon DA et al. who did a mix method study on allied health professionals.^20^ These findings contrast with the finding of a document analysis done on allied health professions accreditation standards for work-integrated learning which identified a gap in the availability of evidence to inform practice and its association with improvement in the quality of care.^21^ These findings were supported by a study done by Murry LT et al. in USA on pharmacist, in which preference was given for practical hands-on training over theory-type activities.^22^

Barriers to CPD include time constraints, busy work schedules, financial constraints, limited investment, limited access, and institutional obstacles aligned with the previous literature.^23^ Furthermore, the experts highlighted the lack of CPD programs and the challenge posed by a large number of allied professionals in Pakistan once CPD activities are planned for them. CPD design, delivery, and limited choice of CPD activities were identified as barriers in scoping review of the literature.^24^ Solutions to these barriers include flexible learning formats; financial assistance; scholarships; allowances from the government; the promotion of distance learning activities; and employer incentives, suggested by the experts of our study and aligned with the finding of Sockalingam S et al. who suggested the use of virtual platform, different teaching approaches, promotion of multi-directional learning and provider support through a community of practice.^25^ The role of governing bodies is crucial in addressing these barriers and considering these factors when planning, designing, and implementing CPD programs. By setting precise standards and criteria, professionals are motivated to continuously improve their skills and knowledge, leading to better patient care outcomes.^25^

The findings of the study on establishing CPD standards and integrating CPD activities into licensing renewal received 79% agreement. This low level of agreement may be attributed to financial involvement in CPD provision and opposed by a study, that adopted CPD as an option for relicensing. In another study performed in Ireland on emergency medical technicians, paramedics, and advanced paramedics, the majority agreed to maintain evidence of CPD activities to ensure registration.^26^

Experts suggest creating a culture of CPD, promoting a positive workplace environment, and providing adequate time for CPD, staffing, and administrative support, as aligned with previous research.^24^,^25^ Solutions include protected time, proper shift allocations, flexibility in working structure, and adequate workforce distribution. The study also revealed 89% agreement for incorporating virtual reality and simulation-based training and skill labs and 93% agreement for job training and e-learning modules, as supported by a study conducted in Sweden with nurses, which highlighted the importance of onsite learning.^2^

Experts suggest that CPD activities should be tailored to the specific needs and interests of AHPs, as they are a diverse group with varying numbers across different countries and settings. While CPD can increase knowledge, it may not necessarily lead to quick changes in clinical practice or improved patient safety.^5^,^23^ A mixed model approach that combines mandatory and voluntary activities is suggested. Regular review and updating of CPD standards can provide stability and reliability.^25^ Both face-to-face and online CPD activities are effective. Regarding the quantity of CPD, there is no direct evidence to suggest, but in practice, complex and less commonly used skills may diminish within four months to one year.^2^,^23^

This study highlights the importance of interprofessional education (IPE) in promoting inclusivity, teamwork, and collaboration among AHPs. It suggests activities such as joint training sessions and team-based learning approaches aligned with the U.S. study with Deans of AHP, who consider IPE as a positive addition.^24^ IPE in allied healthcare can improve patient care quality and safety and enhance healthcare personnel’s collaboration.^24^ The COVID-19 pandemic has also highlighted the benefits of virtual, interprofessional CPD programs for lifelong learning, as suggested by previous studies.^25^ The study also emphasized the need for tailored assessment methods and flexible systems for CPD activities. Various assessment techniques have been suggested, including observational assessments, portfolio reviews, OSCEs, self-assessments, peer reviews, written examinations, 360-degree feedback, simulations, and role-playing. Feedback to regulatory bodies is essential for evaluating CPD effectiveness. However, experts caution against overemphasis on assessment of CPD activities, as it may diminish the intrinsic value of learning and professional development.^26^

This study provides first Consensus-based CPD standards for allied health professionals in the local context of Pakistan. WFME has devised CPD standards for medical doctors globally, but there was a gap in low and middle-income countries for allied professionals. This study aims to fill this gap using a locally adapted Delphi process and provides a practical framework for a large but often overlooked workforce for implementation in Pakistan. The framework supports improved healthcare delivery, staff retention, and professional recognition, and aligns with global quality benchmarks (WFME), making it applicable in both national and international contexts.

### Strengths limitation and future direction:

One of the key strengths of this study is the use of the Delphi method, which facilitated expert-driven consensus across three iterative rounds, thereby enhancing the credibility and validity of the findings. The alignment with WFME 2024 standards gives it a strong international foundation. The diversity of participants and practical focus of the standards also contribute to the robustness and real-world relevance of the study. Future studies should focus on implementing these CPD standards in different institutional settings to assess their feasibility and impact. It is also important to study how these standards influence professional behavior, job satisfaction, and patient outcomes. Further research could explore discipline-specific CPD needs within various allied health fields and how digital platforms can support CPD in remote areas. The study concluded after the third round, once agreement was reached on all the items. While this consensus is valuable, further strengthening could be achieved through qualitative focus group discussions and additional rounds, which were not feasible due to time constraints. Additionally, reliance on a select panel of experts may overlook the input of other stakeholders, such as allied health professionals and faculty.

### Proposed CPD Standards for Allied Healthcare Professionals in Pakistan

### Domain 1: Purposes

### Standard 1.1:

CPD for allied healthcare professionals is guided by a publicly stated purpose aligned with Pakistan’s national health vision and professional development needs. The stated purpose must be reviewed every three years and published on regulator’s website.

Guidance: The purpose highlights knowledge enhancement, skill development, personal growth, improved patient care, and ethical practice. It is developed collaboratively with stakeholders and reviewed every 3 yearly to ensure that it remain current and transparent to all stakeholders.

### Standard 1.2:

CPD standards are implemented consistently across allied health professions and institutions, supporting alignment with national health priorities and institutional professional development goals.

### Guidance:

This includes national coordination and reduction of fragmentation in CPD practices across disciplines.

### Domain 2: Process and Content of CPD

### Standard 2.1:

CPD programs provide flexible, accessible, and relevant learning opportunities tailored to individual and professional needs and requires that at least 30% of activities from self-paced online learning (SOL), distance learning (DL), or structured self-directed learning activities. These activities must be documented in the professional’s CPD portfolio and, where applicable, certified by the provider or supervisor.

### Guidance:

These learning formats enhance flexibility and access, especially for professionals in remote or busy clinical settings. Documentation and verification ensure accountability and recognition of non-traditional learning methods.

### Standard 2.2:

Mandate 20 CPD credits per year, with at least 12 credits in their own field of work, the remaining can be generic CPD activities.

### Guidance:

Regulatory bodies define minimum annual requirements and ensure systems for tracking participation.

### Standard 2.3:

CPD supports development of core professional competencies, including knowledge, clinical and communication skills, critical thinking, decision-making, and ethical practice.

### Standard 2.3a:

CPD supports the development of evidence-base and comprehensive professional knowledge.

### Guidance:

CPD content includes scientific advancements, clinical guidelines, and discipline-specific updates.

### Standard 2.3b:

CPD encourages evidence-base practice to promote safe, and assessment-oriented patient care.

### Guidance:

Hands-on sessions, simulations, and clinical workshops are part of CPD delivery.

### Standard 2.3c:

CPD strengthens communication skills for effective interaction with patients, families, and colleagues.

### Guidance:

Training includes patient-centered communication, teamwork, and cultural sensitivity.

### Standard 2.3d:

CPD fosters critical thinking to enable professionals to analyze, interpret, and respond to complex clinical situations.

### Guidance:

Problem-based learning and case analysis should be integrated.

### Standard 2.3e:

CPD builds decision-making competencies for evidence-based and ethically sound practice.

### Guidance:

Institutions are encouraged to integrate such access into accreditation requirements to support consistent availability

### Standard 2.3f:

CPD promotes ethical awareness and professionalism in healthcare practice.

### Guidance:

Topics include patient rights, confidentiality, informed consent, and professional behavior

### Standard 2.4:

Allied health professionals demonstrate proficiency in the safe operation and handling of clinical equipment and integration of new technologies.

### Guidance:

Skill-based training and hands-on sessions are incorporated into CPD activities.

### Standard 2.5:

CPD systems require institutions to allocate one protected CPD day per quarter (earning a minimum of six CPD credits per day) and offer at least two accredited online modules per year. These measures help quantify institutional support for CPD and address common barriers such as time and access.

### Guidance:

Protected time excludes clinical duties, and modules must be relevant, accredited, and user-friendly.

### Domain 3: Assessment

### Standard 3.1:

CPD activities include assessment methods valid for the relevant learning domain.

### Guidance:

While feedback is a critical component of CPD assessment, it should be complemented by appropriate tools—such as tests, skill evaluations, and reflective exercises—to support meaningful learning and professional growth.

### Standard 3.2:

CPD incorporates individualized formal, constructive and specific feedback which should be formally documented.

### Guidance:

Feedback mechanisms are timely, user-friendly, and contribute to reflective learning.

### Standard 3.3:

individuals maintain digital records and portfolios for CPD tracking and feedback to regulatory authorities to standardize reporting and enables regular oversight of CPD engagement.

### Guidance:

For allied healthcare professionals in Pakistan, each CPD portfolio must include at minimum:

**Date of activity**

*Title/topic of CPD activity*

*Type of activity* (e.g., clinical, academic, publication)

*Credit hours/CPD points awarded*

**Assessment outcome** (if applicable)

*Mode of learning* (in-person/online)

**CPD provider name**

*Participant reflection or learning summary* (optional but encouraged)

Individuals should submit **quarterly electronic reports** to relevant regulatory authorities (e.g., allied councils or the Ministry of Health) summarizing CPD participation, compliance rates, and any feedback from professionals. A centralized, secure digital system should be used to store and manage these records for audit and renewal purposes.

### Domain 4: The Individual Allied Health Professional

### Standard 4.1:

Allied health professionals actively engage in CPD and are supported by employers through protected time, incentives, and recognition.

### Guidance:

It requires employers to offer financial incentives(e.g., bonus point or paid CPD leave) and publish CPD participation rate annually. It aligns with institutional support with measurable rewards and transparency.

### Standard 4.2:

Institutions provide on-the-job training tailored to the specific roles and responsibilities of allied professionals.

### Guidance:

CPD offerings are embedded within clinical and academic work environments.

### Domain 5: Recognition of CPD

### Standard 5.1:

CPD activities—including structured, self-directed, and interprofessional learning—are formally recognized within regulatory and institutional frameworks. One credit equals 1 hour of active face to face learning experience (one hour of lecture should not be counted as 1 CPD point). similarly, 1.5 or 2 hours of engaging online or distance learning can be allocated 1 credit. It require providers to issue digital credit certificate.

### Guidance:

Regulatory bodies assign one credit equals to 1 hour of learning and creates a common currency for CPD and simplifies credit tracking.

### Standard 5.2:

CPD is a mandatory component of professional standards and linked to licensing, accreditation, and renewal of license.

### Guidance:

National and institutional policies clearly integrate CPD into professional pathways for a smooth transition of mid-career professionals and embed CPD into career pathway.

### Domain 6: Educational Resources

### Standard 6.1:

Equitable access to CPD resources is ensured across all geographic and institutional contexts by regulatory body.

### Guidance:

Low-cost, online, and asynchronous learning options are available, especially for rural/remote professionals.

### Standard 6.2:

Simulation-based learning, virtual reality, and skill labs are integrated into CPD delivery, and their implementation is ensured by CPD providers and institutions in alignment with regulatory body guidelines.

### Guidance:

Technology enhances learning in both technical and communication skills.

### Standard 6.3:

CPD content is evidence-based, free from conflict of interest and guided by clearly defined content standards aligned with national and international guidelines.

### Guidance:

Providers disclose sponsorship and maintain educational independence.

### Domain 7: Quality Improvement

### Standard 7.1:

CPD programs undergo continuous evaluation and improvement based on participant feedback and outcome measures.

### Guidance:

Surveys, performance audits, and needs assessments are used to revise and update content.

### Domain 8: Governance and Administration

### Standard 8.1:

A national governance framework and establishment of CQI at institution level ensures oversight, coordination, and strategic direction of CPD for allied health professionals.

Guidance: Roles of professional councils, academic institutions, and regulators are clearly defined.

### Standard 8.2:

Employers and institutions contribute to CPD through the provision of funding, infrastructure, faculty/ experts for CPD and enabling policies and procedures.

### Guidance:

Incentives, dedicated CPD budgets, and integration into human resource development plans are encouraged.

These standards are derived from Delphi expert consensus and aligned with the WFME 2024 CPD framework. They serve as a foundation for national CPD policy development and implementation for allied health professionals in Pakistan.

## CONCLUSION

This study explores health professionals’ perceptions regarding the development of CPD standards for allied healthcare professionals in low- and middle-income countries. It emphasizes regular review, stakeholder involvement, and adaptability to changing needs. The findings establish a foundational framework for CPD standards, but challenges persist in implementation and assessment. Addressing these issues requires collaboration, innovative approaches, and ongoing research.

## Availability of data and material:

The datasets generated during and analyzed during the current study are available through the authors and can be made available upon request.

### Authors’ contributions:

***ST:*** *D*ata curation, formal analysis, investigations and responsible for accuracy of study.

***UM:*** Review and editing of the manuscript.

***KA:*** *P*reparation of the original draft of the manuscript

***RAK:*** review and editing of the manuscript.

All the authors have read and approved the final version of the manuscript.
